# Patient- and Operator-Related Factors Associated With Difficult Thoracic Epidural Catheter Placement: A Retrospective Observational Study

**DOI:** 10.7759/cureus.114927

**Published:** 2026-08-21

**Authors:** Shota Tanimoto, Takuya Mizukami, Ken Hirai, Sae Ono, Tomoharu Shakuo, Yukiko Inoue, Seiji Mochizuki, Takuya Miwa, Masayoshi Kawasaki, Ko Imamura, Atsunori Sakamoto, Megumi Hashimoto, Kenji Shida, Naoki Uchida

**Affiliations:** 1 Department of Anesthesiology, Showa Medical University Northern Yokohama Hospital, Yokohama, JPN; 2 Division of Clinical Pharmacology, Showa Medical University School of Medicine, Tokyo, JPN

**Keywords:** body mass index: bmi, difficult catheter placement, epidural catheterization, operator experience, thoracic epidural anesthesia

## Abstract

Background

Thoracic epidural catheter placement can be technically challenging because of the anatomical characteristics of the thoracic spine. Here, we aimed to identify patient- and operator-related factors associated with difficult thoracic epidural catheter placement, defined as requiring two or more skin punctures.

Methods

In this retrospective observational study, we analyzed the data of consecutive adult patients who underwent thoracic, abdominal, or pelvic surgery under general anesthesia combined with thoracic epidural anesthesia. Patient- and operator-related factors associated with difficult thoracic epidural catheter placement were evaluated using multivariable logistic regression analysis. Difficult placement was defined as requiring two or more skin punctures.

Results

Among 304 consecutive patients who underwent the procedure between June 1 and December 31, 2025, 4 were excluded owing to insufficient documentation, leaving 300 patients for analysis. Difficult placement occurred in 144 patients (48.0%). In the multivariable logistic regression analysis, older age (adjusted odds ratio, 1.02 per year; 95% confidence interval, 1.00-1.04; p = 0.049), higher body mass index (adjusted odds ratio, 1.14 per kg/m²; 95% confidence interval, 1.06-1.22; p < 0.001), and use of the paramedian approach (adjusted odds ratio, 4.94; 95% confidence interval, 2.95-8.45; p < 0.001) were associated with a higher likelihood of difficult placement. More years of anesthesiology experience were associated with a lower likelihood of difficult placement (adjusted odds ratio, 0.89 per year; 95% confidence interval, 0.83-0.95; p < 0.001). Difficult placement was also associated with longer procedure time and greater measured depth to the epidural space. Thoracic puncture level (Th) and a history of spinal disease or surgery were not independently associated with difficult placement.

Conclusions

Older age, higher body mass index, and less operator experience were associated with difficult thoracic epidural catheter placement. Assessing these factors before the procedure may help clinicians anticipate technical difficulties and plan the procedure accordingly. A significant association was also observed between the paramedian approach and difficult placement, but its clinical implication remains uncertain.

## Introduction

Thoracic epidural anesthesia (TEA) may improve postoperative analgesia, promote postoperative recovery, and reduce respiratory complications in patients undergoing highly invasive procedures such as upper abdominal and thoracic surgery. However, TEA is subject to anatomical constraints specific to the thoracic spine, including the steep angulation and overlap of the spinous processes and age-related narrowing of the intervertebral spaces, making the procedure technically more challenging than lumbar epidural anesthesia [1,2]. Difficult epidural catheter placement can increase physical and psychological distress owing to prolonged procedure time and repeated puncture attempts and may also increase the risk of complications such as accidental dural puncture [3-5]. Furthermore, epidural catheter malposition and inadequate analgesia may affect overall perioperative management, resulting in poor postoperative pain control, anesthetic plan changes, and delayed postoperative recovery. Therefore, preprocedural prediction of difficult epidural puncture is clinically important.

Several studies have investigated the factors associated with technical challenges of epidural anesthesia [1-7]. In obstetric practice, pregnant women with obesity are at a higher risk of epidural anesthesia failure and multiple puncture attempts than pregnant women without obesity [3]. A high body mass index (BMI) is also associated with difficult epidural catheter placement, a lower success rate of epidural anesthesia, prolonged procedural time, and delayed recognition of epidural failure [6]. However, practical preprocedural indicators for predicting technically difficult TEA have not been sufficiently established. Therefore, we aimed to identify patient- and anesthesiologist-related factors associated with difficult thoracic epidural catheter placement, defined as requiring two or more skin punctures.

## Materials and methods

In this retrospective observational study, we included consecutive adult patients who underwent thoracic, abdominal, or pelvic surgery under general anesthesia combined with TEA at Showa Medical University Northern Yokohama Hospital between June 1 and December 31, 2025. For TEA, the attending anesthesiologist selected an appropriate thoracic vertebral level according to the type of surgery and performed the procedure using either the midline or paramedian approach. The study was conducted in accordance with the Declaration of Helsinki and Ethical Guidelines for Medical and Biological Research Involving Human Subjects and was approved by the Ethics Committee of Showa Medical University (Approval no. 2025-0252). Informed consent was obtained using an opt-out approach.

Study endpoints

The primary endpoint of this study was the presence or absence of difficult thoracic epidural catheter placement. Difficult placement was defined as requiring two or more skin punctures, consistent with a previous definition of difficult epidural placement as requiring more than one attempt [8]. Data regarding patient characteristics, surgical procedures, and procedural details were collected. Patients’ physical status was assessed using the American Society of Anesthesiologists Physical Status classification (ASA-PS) [9]. As an operator-related factor, years of experience in anesthesiology were recorded and used as a surrogate measure of procedural experience. In cases in which the procedure was taken over by a senior anesthesiologist, the years of anesthesiology experience of the initial operator who performed the puncture attempts before the takeover were used in the analysis. Operators with 0 years of experience in anesthesiology were defined as junior residents; cases in which TEA was performed by anesthesiologists with 0 to 33 years of experience were included.

Obesity was defined as a BMI of ≥ 25 kg/m² according to the criteria of the Japan Society for the Study of Obesity [10]. Obesity was not further graded by severity because BMI was analyzed as a continuous variable in the multivariable analysis. “History of spinal disease/surgery” was defined as the presence of a history of spinal disease such as lumbar disc herniation, spinal canal stenosis, or osteoporosis, or spinal surgery. Osteoporosis was included in this composite variable because vertebral deformity associated with osteoporosis may alter the technical difficulty of epidural anesthesia. Years of experience in anesthesiology were categorized as Junior (0-2 years), Mid-level (3-9 years), or Senior (≥10 years). For descriptive purposes, the thoracic puncture level was categorized as high (Th4-7), middle (Th8-9), or low (Th10-12). In the multivariable analysis, the actual thoracic vertebral level was treated as a continuous variable to evaluate whether procedural difficulty changed progressively with the puncture level while avoiding information loss from categorization. A one-unit increase represented a puncture performed at one thoracic level more caudally. Procedure-related variables included TEA procedure time (Time), number of puncture attempts (Attempt), depth to the epidural space (Depth), thoracic vertebral level of puncture, and the presence or absence of TEA-related complications.

Thoracic epidural catheter placement

Epidural puncture and catheter placement were performed using the Perifix® Standard Kit (NRFit®-compatible continuous epidural anesthesia catheter kit, PSK-S1000RN; B. Braun, Melsungen, Germany), which included an 18-G, 80-mm Tuohy epidural needle. Epidural puncture was performed with the patient in the lateral decubitus position using a Tuohy needle, and entry of the needle tip into the epidural space was confirmed using the loss-of-resistance-to-saline technique. No standardized criteria were established for selecting the midline or paramedian approach; the approach was selected by the operator based on clinical judgment. For the paramedian approach, the needle insertion angle and trajectory were not standardized and were determined by each operator based on clinical judgment and anatomical landmarks. Specific insertion angles were not systematically recorded. Successful epidural anesthesia was defined as successful placement of the catheter in the epidural space. Epidural anesthesia failure included both failure of the needle tip to reach the epidural space and cases in which epidural analgesia was considered inadequate intraoperatively, and the analgesic method was changed to intravenous patient-controlled analgesia (IV-PCA). The decision to switch to IV-PCA was made by the anesthesiologist in charge. When the initial operator determined that reaching the epidural space remained challenging despite multiple puncture attempts, a senior anesthesiologist assumed responsibility for the procedure at the discretion of the supervising anesthesiologist. Each new skin puncture was counted as one attempt, whereas redirection of the needle within the same attempt was not considered an additional attempt.

Statistical analysis

Continuous variables are presented as the mean ± standard deviation or median (interquartile range), as appropriate according to their distributions. For the univariable analysis, age, height, BMI, years of anesthesiology experience, and thoracic puncture level were categorized and compared using Pearson’s χ² test. Depth to the epidural space and procedure time were compared using the Wilcoxon rank-sum test (test statistic: W). Other categorical variables were compared using Pearson’s χ² test (test statistic: χ²) or Fisher’s exact test, as appropriate. Multivariable logistic regression analysis was performed with difficult puncture (Attempts ≥2) as the dependent variable to evaluate independent associations with patient- and operator-related factors, and the Wald test (test statistic: z) was used to evaluate the significance of each predictor. Odds ratios (ORs) and 95% confidence intervals (CIs) were calculated. In the multivariable analysis, age, ASA-PS, BMI, years of experience in anesthesiology, and thoracic puncture level (Th) were treated as continuous variables, whereas sex, history of spinal disease/surgery, and approach were treated as categorical variables. All tests were two-sided, and a p-value <0.05 was considered statistically significant. Statistical analyses were performed using R version 4.5.1 (R Foundation for Statistical Computing, Vienna, Austria).

## Results

A total of 304 consecutive patients who underwent thoracic, abdominal, or pelvic surgery under general anesthesia combined with TEA were enrolled. Of these, 4 patients were excluded from the analysis owing to insufficient documentation, leaving a total of 300 patients for the final analysis.

Baseline characteristics are shown in Table 1. The mean age was 66.6 ± 15.0 years, mean height was 161.7 ± 9.2 cm, and mean BMI was 22.3 ± 4.0 kg/m². Obesity (BMI ≥ 25 kg/m²) was present in 64 patients (21.3%). Regarding procedure-related variables, the mean procedure time was 11.99 ± 6.0 min, the number of puncture attempts was 1.86 ± 1.19, the depth to the epidural space was 5.12 ± 1.15 cm, and the median puncture level was Th9 (Th6-Th10). The most common surgical procedures were colorectal surgery in 113 patients (37.7%), lung surgery in 71 patients (23.7%), and gastric surgery in 37 patients (12.3%).

**Table 1:** Baseline patient and procedural characteristics IQR = interquartile range, SD = standard deviation

Variables	N = 300
Experience (years), Mean ± SD	3.4 ± 5.5
Age (years), Mean ± SD	66.6 ± 15.0
Height (cm), Mean ± SD	161.7 ± 9.2
Weight (kg), Mean ± SD	58.7 ± 13.2
BMI (kg/m^2^), Mean ± SD	22.30 ± 4.00
Time (min), Mean ± SD	11.99 ± 6.00
Thoracic puncture level, Median (IQR)	9.00 (6.00, 10.00)
Attempts, Mean ± SD	1.86 ± 1.19
Depth (cm), Mean ± SD	5.12 ± 1.15
Surgical procedure	
Abdominal	1 (0.3%)
Adrenal	1 (0.3%)
Breast	2 (0.7%)
Chest	3 (1.0%)
Colon	113 (37.7%)
Gallbladder	3 (1.0%)
Kidney	15 (5.0%)
Liver	8 (2.7%)
Lung	71 (23.7%)
Ovary	5 (1.7%)
Pancreas	5 (1.7%)
Rectum	25 (8.3%)
Stomach	37 (12.3%)
Thymus	3 (1.0%)
Uterus	8 (2.7%)
Mean ± SD; Median (Q1, Q3); n (%)	

Difficult puncture (Attempts ≥2), the primary endpoint, was observed in 144 of the 300 patients (48.0%). The clinical success rate of epidural anesthesia was 99.3% (298/300). Of the two failed cases, one was switched to IV-PCA because the epidural analgesic effect was judged to be inadequate intraoperatively, whereas the procedure was terminated without successful identification of the epidural space in the other. A procedure-related complication (accidental dural puncture) occurred in 1 of the 300 patients (0.3%).

The results of the univariable analysis comparing the characteristics of patients with and without difficult puncture are shown in Table 2. Age, sex, height, ASA-PS, and history of spinal disease/surgery were not significantly associated with difficult puncture (p > 0.05). In contrast, a higher BMI was significantly associated with difficult puncture (p < 0.001). Among the 144 patients with difficult puncture, 40 (27.8%) were obese. Years of experience in anesthesiology were also significantly associated with difficult puncture (p = 0.003). Junior operators (0-2 years) accounted for 79.0% of the difficult-puncture group, whereas Senior operators (≥ 10 years) accounted for only 4.2%.

**Table 2:** Univariate analysis of factors associated with thoracic epidural puncture difficulty ASA-PS = American Society of Anesthesiologists Physical Status classification, BMI = body mass index

Characteristic	Overall N = 300^1^	1 attempt N = 156^1^	2 or more attempts N = 144^1^	Test statistic^2^	p-value^2^
Age (years)				0.709	0.702
<40	17 (5.7%)	10 (6.4%)	7 (4.9%)	-	-
40-64	98 (33%)	53 (34%)	45 (31%)	-	-
>=65	185 (62%)	93 (60%)	92 (64%)	-	-
Sex	-	-	-	2.39	0.122
Female	141 (47%)	80 (51%)	61 (42%)	-	-
Male	159 (53%)	76 (49%)	83 (58%)	-	-
Height (cm)	-	-	-	1.53	0.465
<155 cm	74 (25%)	42 (27%)	32 (22%)	-	-
155-164 cm	111 (37%)	59 (38%)	52 (36%)	-	-
>=165 cm	115 (38%)	55 (35%)	60 (42%)	-	-
BMI (kg/m2)	-	-	-	15.6	<0.001
Underweight (<18.5)	48 (16%)	36 (23%)	12 (8.3%)	-	-
Normal (18.5-24.9)	188 (63%)	96 (62%)	92 (64%)	-	-
Obese (>=25)	64 (21%)	24 (15%)	40 (28%)	-	-
ASA-PS	-	-	-	0.536	0.765
1	70 (23%)	39 (25%)	31 (22%)	-	-
2	213 (71%)	108 (69%)	105 (73%)	-	-
3	17 (5.7%)	9 (5.8%)	8 (5.6%)	-	-
Experience (years)	-	-	-	11.7	0.003
Junior (0-2 yrs)	216 (72%)	102 (65%)	114 (79%)	-	-
Mid-level (3-9 yrs)	54 (18%)	30 (19%)	24 (17%)	-	-
Senior (>=10 yrs)	30 (10%)	24 (15%)	6 (4.2%)	-	-
Thoracic puncture level (Th)	-	-	-	0.350	0.840
High (Th4-7)	95 (32%)	48 (31%)	47 (33%)	-	-
Mid (Th8-9)	81 (27%)	41 (26%)	40 (28%)	-	-
Low (Th10-12)	124 (41%)	67 (43%)	57 (40%)	-	-
Approach	-	-	-	40.5	<0.001
Midline	149 (50%)	105 (67%)	44 (31%)	-	-
Paramedian	151 (50%)	51 (33%)	100 (69%)	-	-
History of spinal disease/surgery	21 (7.0%)	13 (8.3%)	8 (5.6%)	-	0.374
Depth to epidural space (cm)	5.00 (4.50, 6.00)	5.00 (4.00, 5.50)	5.50 (4.75, 6.00)	7,595	<0.001
Procedure Time (min)	10.0 (8.0, 15.0)	8.0 (7.0, 10.0)	14.5 (11.0, 19.0)	2,853	<0.001
Epidural Success	-	-	-	-	0.230
Failed	2 (0.7%)	0 (0%)	2 (1.4%)	-	-
Successful	298 (99%)	156 (100%)	142 (99%)	-	-
^1^ n (%); Median (Q1, Q3)					
^2^ Pearson’s Chi-squared test; Wilcoxon rank sum test; Fisher’s exact test. Pearson’s χ² test (χ² statistic) was used for age, sex, height, BMI, ASA-PS, experience category, thoracic puncture level, and approach. Fisher’s exact test was used for history of spinal disease/surgery and epidural success; no conventional test statistic is reported for Fisher’s exact test. The Wilcoxon rank-sum test (W statistic) was used for depth to the epidural space and procedure time.					

The distribution of approaches varied according to the presence or absence of a difficult puncture (p < 0.001). In the difficult-puncture group, the midline approach accounted for 31%, whereas the paramedian approach accounted for 69%. However, thoracic puncture level (Th) was not associated with difficult puncture (p = 0.840). Furthermore, the measured depth to the epidural space was greater in the difficult-puncture group (difficult, 5.50 (4.75, 6.00) cm; non-difficult, 5.00 (4.00, 5.50) cm; p < 0.001), and the procedure time was also longer (difficult, 14.5 (11.0, 19.0) min; non-difficult, 8.0 (7.0, 10.0) min; p < 0.001). The overall clinical success rate of epidural anesthesia was 99.3% (298 patients). Because only two clinical failures (0.7%) occurred, the association between difficult thoracic epidural catheter placement and clinical failure could not be meaningfully evaluated.

The results of the multivariable analysis are shown in Table 3. In the multivariable logistic regression analysis with difficult puncture as the dependent variable, older age (OR: 1.02, 95% CI: 1.00-1.04, p = 0.049), BMI (OR: 1.14, 95% CI: 1.06-1.22, p < 0.001), years of experience in anesthesiology (OR: 0.89, 95% CI: 0.83-0.95, p < 0.001), and the paramedian approach (OR: 4.94, 95% CI: 2.95-8.45, p < 0.001) were independently associated with difficult puncture. In contrast, sex (p = 0.323), ASA-PS (p = 0.210), history of spinal disease/surgery (p = 0.330), and Th (OR: 1.05, 95% CI: 0.93-1.18, p = 0.462) were not independently associated with difficult puncture.

**Table 3:** Multivariable logistic regression analysis of factors associated with thoracic epidural puncture difficulty ASA-PS = American Society of Anesthesiologists Physical Status classification, CI = confidence interval, OR = odds ratio

Characteristic	Odds Ratio	z-value1	95% CI	p-value
Age (years)	1.02	1.97	1.00, 1.04	0.049
BMI (kg/m2)	1.14	3.63	1.06, 1.22	<0.001
Sex				
Male	—	—	—	
Female	0.76	-0.989	0.44, 1.31	0.323
ASA-PS	0.69	-1.25	0.38, 1.23	0.21
History of spinal disease/surgery				
No	—	—	—	
Yes	0.41	-0.973	0.05, 2.20	0.33
Experience (years)	0.89	-3.32	0.83, 0.95	<0.001
Approach/Method				
Midline	—	—	—	
Paramedian	4.94	5.97	2.95, 8.45	<0.001
Thoracic Puncture Level (Th)	1.05	0.736	0.93, 1.18	0.462
1 Wald test z-statistic				

## Discussion

This study investigated factors associated with difficult thoracic epidural catheter placement, defined as two or more skin punctures, in patients who underwent TEA for thoracic, abdominal, or pelvic surgery. The results showed that older age, higher BMI, and fewer years of operator experience were associated with difficult puncture. A significant association was also observed between the paramedian approach and difficult puncture. Meanwhile, Th and history of spinal disease/surgery were not independent factors associated with difficult puncture, at least in the present study population. In addition, patients with difficult puncture had longer procedure time and greater measured depth to the epidural space.

The finding that higher BMI was associated with difficult puncture was consistent with previous reports. We found that BMI was an independent factor associated with difficult puncture (OR 1.14, 95% CI 1.06-1.22, p < 0.001); moreover, the measured depth to the epidural space was greater in the difficult-puncture group (difficult, 5.50 cm; non-difficult, 5.00 cm; p < 0.001). Previous studies have demonstrated that in patients with obesity, the distance from the skin to the epidural space is increased [7,11,12], and that this increase is associated with an increased risk of multiple puncture attempts [3,4,6]. Older age was also associated with difficult puncture (OR: 1.02, 95% CI: 1.00-1.04, p = 0.049). In older patients, age-related narrowing of the interspinous and interlaminar spaces, ossification of the ligaments, and hypertrophy of the facet joints act in combination, making needle advancement into the interlaminar space more challenging [13]. A 3-D computed tomography (CT) study of TEA revealed that the number of occluded interlaminar spaces in the mid-thoracic spine and ossification of the supraspinous ligament were associated with puncture success, the number of attempts, and procedure time [2], suggesting that age-related morphological changes in the thoracic spine affect puncture difficulty. Although previous studies have identified obvious spinal deformity as a predictor of difficult neuraxial puncture [14-16], history of spinal disease/surgery was not an independent factor in the present study.

Regarding operator experience, fewer years of experience were associated with a higher frequency of difficult puncture. Years of experience in anesthesiology were independently associated with difficult puncture (OR 0.89, 95% CI 0.83-0.95, p < 0.001), and the odds of difficult puncture decreased as the number of years of experience increased. Consistent with these findings, operator experience has been associated with first-attempt success and the number of attempts [17]. Regarding the puncture approach, we observed an association between the paramedian approach and difficult puncture (OR, 4.94; 95% CI, 2.95-8.45; p < 0.001), whereas a previous study reported no clear association between procedural difficulty and the use of the midline or paramedian approach [14]. Therefore, our finding should be regarded as an association observed in this study population rather than evidence that the paramedian approach increases procedural difficulty. No association was observed between Th and difficult puncture in the present study (OR: 1.05, 95% CI: 0.93-1.18, p = 0.462). Previous findings regarding the association between Th and procedural difficulty have been inconsistent. One study reported that puncture at Th1-2 was easier than puncture at Th5-6 [18]. Anatomically, lower thoracic epidural puncture may resemble lumbar epidural puncture and may be less technically demanding [19], whereas mid-thoracic puncture is often considered more challenging [20].

Procedure time was significantly longer in difficult cases (difficult, 14.5 min; non-difficult, 8.0 min; p < 0.001). A previous study of lumbar epidural anesthesia reported that prolonged puncture time and an increased number of attempts were associated with an increased incidence of complications such as accidental dural puncture [5]. Therefore, prolonged puncture time is a procedural indicator that cannot be overlooked when considering the procedural burden on patients. Because procedure time and measured depth to the epidural space are determined during the procedure, these variables should be interpreted as procedural correlates of difficult placement rather than as preprocedural predictors.

This study highlights the clinical importance of assessing the risk of difficult thoracic epidural catheter placement from both patient- and operator-related perspectives. Assessing preprocedural factors associated with difficult puncture may provide useful information for anticipating technical difficulty, reducing the procedural burden on patients, and ensuring safer perioperative management. In particular, patients of advanced age or with a high BMI, anticipating increased procedural challenges before puncture and considering the involvement of an experienced operator or an early change in strategy may help avoid multiple puncture attempts and prolonged procedure time. Furthermore, additional assessment using preoperative CT images to evaluate spinal morphology or ultrasonography to identify the interlaminar space may be useful for reducing procedural difficulty [2,20].

Limitations

First, this was a retrospective observational study conducted at a single institution, and its external validity is limited. However, this study exploratorily investigated the characteristics of difficult-puncture cases based on data obtained in actual clinical practice and may serve as a basis for future prospective studies.

Second, because the approach was selected at the operator’s discretion, confounding by indication and selection bias could not be excluded. The paramedian approach may have been selected from the outset in patients in whom midline puncture was expected to be difficult. In addition, cases in which the initial approach was changed from the midline to the paramedian approach owing to procedural difficulty may not have been completely identified in this retrospective study. Therefore, the association between the paramedian approach and difficult thoracic epidural catheter placement should be interpreted cautiously, as it does not establish a causal relationship.

Third, the assessment of spinal conditions was based on medical history documented in the medical records and did not quantitatively reflect the presence or severity of spinal deformity. Preoperative CT images and ultrasonographic findings were not systematically evaluated. Therefore, we could not directly examine the associations between objective anatomical abnormalities, such as vertebral deformity, narrowing or obstruction of the interlaminar spaces, and ligament ossification, and the difficulty of thoracic epidural catheter placement. In addition, the small number of patients with documented spinal disease or prior spinal surgery may have limited the statistical power to detect an independent association. Fourth, residual or unmeasured confounding cannot be excluded because of the retrospective observational design. In addition, procedural difficulty was defined solely according to the number of skin punctures. Although this definition provides an objective and reproducible endpoint, it may not capture all dimensions of technical difficulty, such as repeated needle redirection within the same skin puncture or other procedural challenges.

## Conclusions

In this retrospective analysis of 300 TEA cases, older age, higher BMI, and fewer years of operator experience were linked to difficult thoracic epidural catheter placement. A significant association was also observed between the paramedian approach and difficult placement, but the clinical implication of this association remains uncertain. Difficult placement was associated with longer procedure times and greater measured depth to the epidural space, whereas no independent association was observed between Th and difficult thoracic epidural catheter placement. Assessing preprocedural factors associated with difficult puncture may help clinicians anticipate technical difficulty, reduce procedural burden on patients, and improve procedural planning.
